# Adaptation and Validation of the Sexuality Attitudes and Beliefs Scale for the Italian Context

**DOI:** 10.3390/ijerph192114162

**Published:** 2022-10-29

**Authors:** Cinzia Gradellini, Shaniko Kaleci, Margarida Sim-Sim, Hélia Dias, Daniela Mecugni, Vicki Aaberg, Sagrario Gómez-Cantarino

**Affiliations:** 1 Campus Universitario San Lazzaro, University of Modena and Reggio Emilia, Vía Amendola, 2-42122 Reggio Emilia, Italy; 2 Università degli Studi di Modena e Reggio Emilia, Vía Amendola, 2-42122 Reggio Emilia, Italy; 3 Comprehensive Health Research Centre Integrated Researcher, Nursing Department, University of Evora, 7000-811 Evora, Portugal; 4 Superior School of Health, Quinta do Mergulhão Srª da Guia, 2005-075 Santarém, Portugal; 5 Faculty of Physiotherapy and Nursing, Toledo Campus, University of Castilla-La Mancha, Avenida Carlos III s/n, 450071 Toledo, Spain; 6 Health Sciences Research Unit, Nursing (UICISA: E), Nursing School of Coimbra (ESEnfC), 3001-901 Coimbra, Portugal

**Keywords:** sexuality, health, care, nurse, evaluation, health education, health promotion

## Abstract

Background: Nurses dealing with a patient’s sexuality must start from an awareness of their own experience, specific attitudes, and possible limits. What emerges from the literature is a conservative tendency in nurses, which underlines the difficulty in this awareness, but even a difficulty in improving the necessary knowledge/skills. It is, therefore, essential to create tools that can raise awareness of these limits. Objective: the present study aims to explore the psychometric properties of the Attitudes and Beliefs about Sexuality Scale, adapted and validated for the Italian context. Method: This is an instrumental, cross-sectional piece of research, whose SABS validation process applies the steps of Beaton and Valmi. The convenience sample collected data from 223 participants in the first approach. This was followed by a retest involving 44 students randomly selected from those who responded in the first phase. Ethical principles were respected. Results: The SABS questionnaire demonstrated good test-retest reliability, good internal consistency, and adequate construct validity. Conclusions: The Italian version of the SABS is valid and reliable for use with nursing students. This is the fourth language in which the SABS is available for research.

## 1. Introduction

The complexity of sexuality assessment stems from the different forms of sexual orientation, which may not correspond to biological sexuality. In the holistic person-centered approach, nurses must understand how people perceive their own sexual identity and role concerning specific health problems [1,2,3], considering that sexual health is an integral part of nursing care [4,5].

In the mentioned holistic approach to patient care, the World Health Organization (WHO) recommends considering the sexual dimension because it is strongly related to personal integrity, body image, and self-identity [6].

Indeed, as a human dimension, sexuality accompanies all moments of life and characterizes social interactions. In Maslow’s hierarchy theory, which describes the order of priority of human needs, sexuality appears at the first level of biological needs. Moreover, sexuality could be considered as an expression of the human being and of the person’s identity in relation to others. In fact, in an affective relationship, sexuality expresses identity and personality, while in a social relationship, it defines the role [6]. The WHO states that sexual health is “fundamental to overall health and well-being” and requires a positive and respectful approach, along with “the possibility of having pleasurable and safe sexual experiences, free of coercion, discrimination, and violence” [7]. 

Considering the above elements, it seems clear how sexuality becomes a psychosocial determinant of health that needs to be assessed and managed [5,8]. Sexual assessment and care require a high level of competence, including (1) counselling skills; (2) in-depth knowledge of risk factors and available treatments; (3) ability to promote prevention of sexually transmitted diseases; (4) skills in sexual dysfunction care and care for victims of sexual violence [8]. Becoming competent in taking charge of sexuality means knowing anatomy, physiology, and biochemistry, but also knowledge about identity, social roles and relationships, and the complexity of emotional aspects [9]. The literature shows that working with health professionals to implement specific competencies to take charge of the sexual dimension is essential to reduce possible barriers. This necessity increases concerning the lesbian, gay, bisexual, transgender, questioning, intersex, and two-spirit (LGBTQI2S) population [10,11], as identity and sexual health specifics are topics that are absent or poorly covered in basic training. Specific contents about queer, intersex, and two-spirit health and wellness were not identified in any medical and nursing curriculum [11]. Moreover, the average time spent on the topic is only two hours [9,12]. No studies report timings in nurse practitioner curricula [13]. What have emerged as the main barriers to these shortcomings are the perceived lack of specific preparation [14] and the difficulty of the lecturers in dealing with the topic [12].

Mainly in Western countries, sexuality seems to be losing its taboo, not least because of the work carried out by the media. On the one hand, this makes it possible to overcome the old taboos; on the other hand, it increases the risks of spreading information, which may be incorrect, incomplete, and contradictory [15], especially for young people. Analysis of media literacy on the subject is absent, although its impact on the youth population is widely recognized. Educational programs that include a deconstruction of media messages, along with work on awareness of how the media influence identity dynamics on sexuality in adolescents, were of great importance [9].

Although sexuality care is fundamental, it is not easy to handle, firstly because it concerns intimate aspects of life, and secondly because it is strongly related to the personhood of human beings. Sometimes, social systems themselves become barriers to sexual and identity well-being as they lead to discrimination in terms of gender and sexual orientation, impacting sexual development, but also emotional development and social relationships [9].

There is no standard of reference, considering that sexual characteristics and needs are extremely individual [16]. In fact, the complexity of sexuality assessment starts from the different forms of sexual orientation, which might not correspond to biological sexuality. In the holistic approach to the person, the nurse must understand how people perceive their sexual identity and role, even considering specific health problems [1]. 

Nurses dealing with patient sexuality must start from an awareness of their own experience, specific attitudes, and possible boundaries [1,2]. What emerges from the literature is a conservative tendency in nurses, which underlines the difficulty in this awareness, but even difficulty in improving the necessary knowledge/skills [2]. The above themes indicate that the creation of tools that can raise awareness of these limits becomes essential.

The Sexual Attitudes and Beliefs Survey (SABS) is a useful self-reported tool to measure how nurses or nursing students feel when dealing with patients’ sexuality. The SABS is a validated scale measuring nurses’ attitudes and beliefs, designed, first in an English version [17], and then translated into Portuguese [2] and Spanish [18]. It assesses nurses’ attitudes and beliefs in caring for their patients’ sexuality and asks for self-administration. The scale consists of 12 items, presented on a six-position Likert scale, ranging from 1 (strongly disagree) to 6 (strongly agree). Items 1, 2, 4, 6, 8, 10 and 12 are reversed. The score can range from 12 to 72, through addition, where a higher score means more barriers to coping with the patient’s sexuality. In the original study, Cronbach’s alpha coefficient was 0.75 and 0.82 in two different assessments 740 [19,20,21].

Considering the relationship between Portuguese and Italian (both neo-Latin languages), an Italian version of the Portuguese scale has been developed and validated in collaboration with the authors themselves. 

The present study addressed the following research question, which measures nurses’ attitudes and beliefs about sexual attitudes: does the SABS have psychometric qualities suitable for the educational context of Italian nursing students? 

The outcome of the study is the Italian linguistic and cultural adaptation and validation of the Portuguese version of the SABS scale.

## 2. Materials and Methods

### 2.1. Study Design

The present study consists of instrumental research, with a cross-sectional approach, validating the SABS scale for Italian [22].

### 2.2. Participants

The conventional sample included all nursing students in all three years of an Italian nursing course (70 first year; 60 second year; 93 third year). The second administration to the same cohort involved 44 second year students who were part of the first-year sample in the first administration. 

Any exclusion criteria have been defined: all nursing students in the above cohorts were eligible to participate if they agreed.

The recommendations followed on the scope of the sample are a minimum of two subjects per item, with an absolute minimum of 100 to 250 subjects [23]. 

The sample consisted of 223 nursing students. Among them, 44 have been pre-selected for re-testing, one year after the first administration. The Italian SABS items were administered to the recruited students from 2 February to 20 February 2019. The response rate of the first data collection was 97.3%. The questionnaire included characterization data (gender, age, year of the course, and involvement in sexual activity). Most of the participants were in the third year of nursing education. In the sample, seventy-six percent (n165) were female. Sixty-six percent (*n* = 144) lived at home with their parents. In terms of gender identity, 84% reported being heterosexual and about 55% (*n* = 119; 54.8%) have only one sexual partner (Table 1).

### 2.3. Procedures

The used methods to first create then validate the Italian version of the SABS scale are from Beaton and Valmi [24,25]. 

In the first phase (creation), the following steps were carried out:

I step: The Portuguese version of the scale was translated from two bilingual people (Portuguese/Italian): one familiar with the colloquial sentences and one knowledgeable about the survey’s contents. The translations were made individually, in a blind study, using a report to specify uncertainties and specific reflections which accompany the definition of two different Italian versions of the survey (T1 and T2) [24].

II step: the translators worked together to create a unique integrated tool (T12).

III step: the integrated Italian version was translated in Portuguese, once again in blind, producing the version O1 and O2 [24,25]. 

IV step: This was divided in 2 different phases: in the first phase, the five versions of the surveys, and the related reports, were evaluated by a team of experts, who defined the first version of the Italian SABS (S1). 

In the second phase (validation), the factor analysis of the S1 version was made, evaluating reliability, test-retest reliability, internal consistency of items, and exploratory factor analysis (EFA).

The study authors obtained permission to translate the Portuguese version (main author participated in this study). The study formed part of a multicenter research project. The University of Modena and Reggio Emilia’s Degree Program Board (in the session of 20 May 2020) and the Ethical Committee of the University of Evora approved the study (registration number 18175).

### 2.4. Measurements

Participation was voluntary. A paper version was prepared and administrated to the students. The paper version was collected in a box to guarantee anonymity. The survey itself includes a letter of presentation and a request for anonymous consent.

The data collection form was the S1 version of the SABS. As previously mentioned, it is introduced by presenting the evaluation’s aims and the request for consent. It has 89 items in total; it is divided into four sections: (A) Socio-family data (29 items); (B) Attitudes and beliefs about sexuality (14 items); (C) Sexuality and sex education behavior questionnaire (17 items); (D) Sexual life quality (29 items: 11 for males, 18 for females). Items are multi-options and close answers, 58 using a 1 to 6 Likert scale (1, strongly disagree; 6, strongly agree). The final score evaluates the answers using a 13-point Survey Qualification Key, which indicates how to read the single data/sections.

### 2.5. Data Analysis

Statistical analyses were performed in STATA version 14 for Windows (Stata Statistical Software: Release 14). Descriptive statistics were performed for demographic variables. Descriptions of variables were made using tables of frequencies, means, and standard deviations (SD). The level of significance was established for *p*-values less than or equal to 0.05.

To assess test-retest reliability, a randomly selected subgroup of the sample was tested twice. The test-retest reliability was assessed by calculating the intraclass correlation coefficient (ICC). The ICC is defined as follows: between-subject variability ÷ (between-subject variability + error); as the error term decreases, the ICC goes from 0 to 1, indicating perfect reliability. There is no universal consensus on how the magnitude of the ICC should be interpreted; Fleiss [25] proposed a classification for the strength of test-retest reliability based on the ICC as follows: < 0.40 poor, 0.40–0.75 fair to good and > 0.75 excellent [26]. Absolute reliability refers to the degree to which repeated measurements of the same instrument on the same individual vary around the valid score; the smaller the variation in repeated measurements, the higher the absolute reliability [27]. Absolute reliability was measured by the standard error of measurement (SEM). The SEM and ICC of a test are inversely related; if a test has perfect reliability, ICC = 1.0, the SEM will be zero, indicating no measurement error [28].

To measure reliability, Cronbach’s alpha calculation performed an internal consistency analysis to assess the reliability of the SABS score. Alpha values were interpreted according to Kline: acceptable (0.60–0.70); good (0.70–0.90); and excellent (≥0.90). In addition, item-test correlations were calculated for each item. For a reliable scale, these values should be greater than 0.30.

Internal consistency assesses the degree to which the items of a test are interrelated. Internal consistency was measured by Cronbach’s alpha. The alpha ranges from 0 to 1; high alpha values indicate a high degree of interrelatedness among the items of a test. Alpha values between 0.70 and 0.95 are considered good. It is important to note that the number of items influences the alpha: the more items in a test, the higher the alpha. In addition, care should be taken when interpreting alpha values higher than 0.90, as this may indicate the presence of redundant items [19]. Internal consistency was calculated for the entire SABS and for each subscale.

An exploratory factor analysis was conducted to determine how the SABS items correlated with each other and to determine whether items could be grouped into subscales. In addition, a preliminary Kaiser-Meyer-Olkin (KMO) test was conducted to determine sampling adequacy; data sets with KMO > 0.5 are considered acceptable for EFA [20]; and the Kolmogorov-Smirnov test was carried out for normality of distribution (*p* > 0.05).

Participants in the validation process were 217, considering the number of items in the scale [22].

## 3. Results

### 3.1. Reliability and Validity

A total of 44 of the 217 included individuals were submitted to test-retest reliability procedures. The test-retest reliability was analyzed through the intraclass correlation coefficient (ICC), whose ranges are shown in Table 2. The excellent tool’s stability is also evident in minimal differences between the average of the questionnaire at T0 and T1, as shown in the same Table 2.

### 3.2. Internal Consistency

The internal consistency was calculated on all 217 included individuals. Cronbach’s alpha coefficient of less than 0.76 was seen for the B: Attitudes and beliefs about sexuality subscale only, while for the total and the three subscales (A: Social-family data (What is your level of family satisfaction?), C: questionnaire of behaviors relating to sexuality and sexual education, D: Quality of sex life:), (Table 3).

The internal consistency of the entire questionnaire was good (alpha = 0.76). The quality of sexual life male group subscale had the highest internal consistency (alpha = 0.97), followed by the quality of sexual life female group subscale (alpha = 0.91). Group C (Questionnaire of behaviors relating to sexuality and sexual education subscale) and B (Attitudes and beliefs about sexuality subscale) had the lowest internal consistencies (respectively, alpha = 0.75 and alpha = 0.64) (Table 4).

### 3.3. Exploratory factor analysis (EFA)

The Kaiser-Meyer-Olkin (KMO) measure of sampling adequacy was 0.70. Datasets with KMO > 0.50 are considered acceptable for EFA [20]. Therefore, we did not proceed with the EFA.

## 4. Discussion

The objectives of this study were the translation, cultural adaptation, and validation of the Sexuality Attitudes and Beliefs Scale (SABS) for the Italian context. The translation of the original SABS was carried out using internationally recognized methods.

### 4.1. Discussion Reliability

The ICC was calculated to obtain the test-retest reliability, and its values were statistically significant, from ICC = 0.41 to ICC = 0.72: it means that the questionnaire has fair stability. The stability evaluation is also reinforced by the minimal differences between the total and partial average of the questionnaire at T0 and T1: it reveals very little change over time in absolute scores. 

The questionnaire has a moderate test-retest reliability: giving the same test to the same individuals on two occasions, its results are consistent over time.

### 4.2. Discussion Internal Consistency 

Cronbach’s alpha coefficient shows acceptable internal consistency only for the sexual quality of life (D) subscale (α = 0.97 for men and 0.91 for women) to consider it the most sensitive for measuring the sexual quality of life.

The scale is especially significant in promoting holistic care [1,2,3]. It could call the professional to pay more attention to the assessment of sexuality (e.g., to devote time, to be open to active listening, to be available for related arguments). It is necessary to remember that sexuality is a lifelong companion, even during illness, and its outcomes are fundamental to overall well-being [7]. The scale measures not only perceived specific competencies [28] (e.g., I am confident in my ability to address concerns about sexual life with patients), but even possible difficulties, creating awareness of one’s own difficulties (e.g., I feel embarrassed to talk about specific arguments).

Considering that sexuality refers to intimate aspects of life [10], it requires professionals to be self-aware in the first place. The scale has two sections, focusing on the conservative tendency [2].

Cronbach’s alpha coefficient shows excellent internal consistency only for the subscale of socio-family data (What is your level of family satisfaction?) (α = 0.84) to consider it the most sensitive for measuring the level of community integration in healthy people. This result is very similar to that of other studies and validations, especially those of the Portuguese version of the SABS. Therefore, we can affirm that the subscale of socio-family data (What is your level of family satisfaction?) has the most significant sensitivity for measuring individual satisfaction in family life; the related items are: I am satisfied with the help I receive from my family at all times; I am worried about something; I am satisfied with the way issues of common interest are discussed in the family and the way problem solving is shared; My family supports my desire to undertake new business or change my lifestyle. I am satisfied with the way my family shows affection and reacts to my feelings, whether they are irritation, pain, or love. I am satisfied with the time I spend with my family).

Becoming competent in taking charge of sexuality means knowing anatomy, physiology, and biochemistry, but also knowledge about identity, social roles and relationships, and the complexity of emotional aspects. New content such as gender diversity and violence, sexual and reproductive health, sexually transmitted diseases, but also emotional literacy, must update in educational curricula [9,29]. Specific topics, related to the LGBTQI2S population in educational programs, would better prepare practitioners to provide inclusive, evidence-based, and person-centered care. Not only that, it would improve the understanding of health problems, enable work on inequalities, and lower barriers to accessing services [30]. Sometimes, nurses’ sexual perception affects attitudes toward care. The sexual self-schema affects feelings, even beliefs and attitudes towards sexuality. This means nurses with a positive sexual self-schema could have more positive attitudes toward patients’ sexuality [3].

Courses regarding sexuality should be included in the nursing curriculum, but it is also important to analyze students’ attitudes towards sexual care [31], to better work on the related didactic offer. Awareness of those findings could promote changes in nursing education, decrease sexual health barriers, and prepare faculty, students, and nurses to provide sexual health care across the lifespan [4]. An extensive review of the literature on educational provision reports that positive results in terms of creating inclusive contexts have not been achieved by traditional lectures, but by social studies that stimulate discussion and confrontation [9]. Another aspect to be considered is that the topic of sexuality is one of the great absentees in terms of evaluating the educational impact on the educational provision.

All these elements underline the need for greater attention and investment in sexual health in educational pathways, but for these to be effective, it is necessary to work from the awareness and evaluation of this awareness that creates new knowledge. Considering the complexity of sexuality in all its dimensions, there is a need for a specific scale to accompany students and teachers in the evaluation of this delicate but at the same time fundamental pathway for future health professionals.

## 5. Conclusions

The Italian version of the SABS is fully validated, demonstrating good test-retest reliability, good internal consistency, and adequate construct validity in nursing students. 

The current study provides a fourth language version, in which the SABS can be made available for culture research. In fact, after the original [17], two more languages, such as Portuguese [2] and Spanish [18], paid attention to the instrument. Following the correct validation process, having the same instrument in different languages is a mechanism that enhances the instrument and, at the same time, enables multicenter studies [32]. 

## 6. Limitation

Even if the sample size is suggested from the literature to complete the validation, qualitatively, a randomized and multicenter sampling (instead of convenience sampling) could give greater value to the results.

## 7. Future Research

The implications for future research are the potential which a validation process can give to the instrument and acceptancy by other researchers, developing knowledge about patient sexuality.

## Figures and Tables

**Table 1:** Characteristics of participants.

	All Participants (N = 217)	
Participant Characteristics	*n*	%
4. Gender		
2. Male	48	22.1
1. Female	165	76
Missing	4	1.8
5. Year of study course		
1° Year	71	32.7
2° Year	60	27.6
3° Year	85	39.2
Missing	1	0.5
6. In the previous academic year, where did you live?		
1. At the parents’ home	144	66.4
2. In university residence	5	2.3
3. In a rented room	11	5.1
4. In a shared rented house	18	8.3
5. Other	14	6.5
Missing	25	11.5
7. Where do you currently live?		
1. At the parents’ home	126	58.1
2. In university residence	6	2.8
3. In a rented room	17	7.8
4. In a shared rented house	26	12
5. Other	23	10.6
Missing	19	8.8
8. If you express sexual orientation, how would currently defined:		
1. Heterosexual	182	83.9
2. Homosexual	5	2.3
3. Bisexual	7	3,2
4. Not yet defined	3	1.4
Missing	20	9.2
9. Affective-sexual situation		
1. I have a single sexual partner	119	54.8
2. I have a sexual partner, but I also have relationships with others	5	2.3
3. Right now I have multiple sexual partners	5	2.3
4. Right now I have no sexual partner	49	22.6
5. I have never had sexual partners	16	7.4
Missing	23	10.6

**Table 2:** Intraclass Correlation Coefficient (ICC) for reliability of the Italian version of the SABS.

		Test (T0)	Re-Test (T1)	ICC
		Mean (SD)	Mean (SD)	(T1-T2)
A: Social-family data (What is your level of family satisfaction?)		1.6 (0.4)	1.7 (0.3)	0.61
B: Attitudes and beliefs about sexuality		3.2 (0.5)	3.1 (0.5)	0.62
C: Questionnaire of behaviors relating to sexuality and sexual education.		2.3 (0.4)	2.0 (0.4)	0.72
D: Quality of sex life:	Male	4.8 (1.4)	2.7 (1.9)	0.43
	Female	4.3 (0.7)	2.5 (1.4)	0.41

**Table 3:** Cronbach’s alpha for internal consistency of the Italian version of the SABS.

		Number of Items	Cronbach’s α	
A: Social-family data (What is your level of family satisfaction?)		5	0.84	
B: Attitudes and beliefs about sexuality		12	0.64	
C: Questionnaire of behaviors relating to sexuality and sexual education.		17	0.75	
D: Quality of sex life:				
	Male	11	0.97	
	Female	18	0.91	

**Table 4:** Descriptive statistics of SABS items.

			% of Subjects Who Were	
A: Social Data	Media (SD)	Min-Max	0 = Almost Never	2 = Almost Always
A10APGAR1	1.72 (0.55)	0-2	4.6	70.5
A10APGAR2	1.51 (0.68)	0-2	9.2	54.8
A10APGAR3	1.72 (0.52)	0-2	3.2	67.3
A10APGAR4	1.63 (0.58)	0-2	4.6	61.8
A10APGAR5	1.68 (0.56)	0-2	4.1	65.9
B: Attitudes and beliefs about sexuality.			1 = Strongly disagree	6 = Strongly agree
B1SABS1	3.62 (1.35)	1-6	6.0	10.6
B1SABS2	4.7 (1.3)	1-6	1.8	36.4
B1SABS3	2.85 (1.55)	1-6	27.2	4.1
B1SABS4	2.97 (1.3)	1-6	12.9	4.6
B1SABS5	3.4 (1.45)	1-6	10.1	9.7
B1SABS6	2.66 (1.33)	1-6	20.3	3.2
B1SABS7	2.88 (1.43)	1-6	18.4	5.5
B1SABS8	3.67 (1.32)	1-6	5.1	11.1
B1SABS9	2.67 (1.43)	1-6	24.4	5.1
B1SABS10	4.03 (1.43)	1-6	5.1	19.4
B1SABS11	3.6 (1.5)	1-6	9.7	13.4
B1SABS12	2.81 (1.25)	1-6	16.1	2.8
C: Behaviors related to sexuality and sexual education			1 = Strongly disagree	5 = Strongly agree
C1AtSex1	2.13 (1.2)	1-5	40.1	4.1
C1AtSex2	1.73 (0.94)	1-5	47.5	1.4
C1AtSex3	2.35 (0.98)	1-5	20.7	1.8
C1AtSex4	1.66 (0.94)	1-5	51.2	2.3
C1AtSex5	2.34 (1.22)	1-5	31.3	3.7
C1AtSex6	2.06 (1.08)	1-5	36.4	2.3
C1AtSex7	1.77 (0.93)	1-5	45.2	0.9
C1AtSex8	2,14 (1,19)	1-6	37.8	0.5
C1AtSex9	1.8 (0.97)	1-5	42.9	2.3
C1AtSex10	1.83 (1.01)	1-5	45.2	1.4
C1AtSex11	1.56 (0.82)	1-5	54.8	0.5
C1AtSex12	1.89 (1.02)	1-5	41.5	1.4
C1AtSex13	2.05 (0.96)	1-4	33.2	6.5
C1AtSex14	4.31 (0.91)	1-6	1.8	0.5
C1AtSex15	3.95 (1.07)	1-5	3.7	32.7
C1AtSex16	4.75 (0.54)	3-5	-	72.4
C1AtSex17	1.72 (0.85)	1-5	45.2	0.5
D: Sexual Life Quality				
For men (women should jump to the next group)			1 = Strongly Agree	6 = Strongly Disagree
QVidaH1	4.75 (1.66)	1-6	0.9	13.8
QVidaH2	4.79 (1.53)	1-6	0.9	12.4
QVidaH3	4.63 (1.89)	1-6	2.8	13.8
QVidaH4	4.78 (1.65)	1-6	1.8	12.9
QVidaH5	4.98 (1.59)	1-6	1.8	14.3
QVidaH6	4.9 (1.63)	1-6	1.8	13.4
QAVidaH7	4.92 (1.51)	1-6	1.4	12.9
QVidaH8	4.98 (1.5)	1-6	1.4	12.9
QVidaH9	5.06 (1.62)	1-6	1.8	15.7
QVidaH10	4.88 (1.69)	1-6	1.8	13.4
QVidaH11	4.62 (1.74)	1-6	1.8	11.5
For women			1 = Strongly Agree	6 = Strongly Disagree
QVidaM1	2.16 (1.57)	1-6	33.2	7.8
QVidaM2	5.22 (1.34)	1-6	3.2	47.5
QVidaM3	5.34 (1.36)	1-6	4.1	53.3
QVidaM4	5.47 (1.3)	1-6	4.1	59.0
QVidaM5	2.54 (1.74)	1-6	28.1	9.2
QVidaM6	5.36 (1.39)	1-6	4.1	55.8
QVidaM7	5.19 (1.37)	1-6	2.8	48.8
QVidaM8	5.4 (1.36)	1-6	4.6	55.3
QVidaM9	2.54 (1.82)	1-6	30.0	12.0
QVidaM10	5.01 (1.47)	1-6	3.7	43.3
QVidaM11	5.39 (1.38)	1-6	3.7	56.2
QVidaM12	4.87 (1.66)	1-6	5.1	42.9
QVidaM13	2.26 (1.73)	1-6	37.3	8.8
QVidaM14	5.11 (1.5)	1-6	5.1	46.1
QVidaM15	5.4 (1.33)	1-6	2.8	55.3
QVidaM16	4.59 (1.77)	1-6	6.5	36.4
QVidaM17	5.25 (1.42)	1-6	4.1	49.8
QVidaM18	3.04 (1.85)	1-6	18.9	13.4

## Abbreviations

WHO	World Health Organization
SABS	Sexuality Attitudes and Beliefs Scale
EFA	Exploratory Factor Analysis
KMO	Kaiser-Meyer-Olkin
